# Development and Implementation of an Antenatal Care Follow-Up Passport at Almanagil Teaching Hospital: A Departmental Initiative in Obstetrics and Gynecology

**DOI:** 10.7759/cureus.97202

**Published:** 2025-11-19

**Authors:** Abdelrahman Edris Osman Ali, Alaa Jamal Mohamed Alaa Jamal Mohamed Ali, Marwa Abdelrazig Eljack Ibrahim, Khadeja F Abdallah, Sara Isam Eldin Ibrahim Elshafie, Mehira Abdelgader Abdalla Abdelmajeed, Doha Abdelbage Suliman Abdelkareem, Hafsa Omer Hassan Elbashir, Rayan Tajelser Mohammed Abdalla, Sara Abdalla Osman Mohamed, Rghada Khalil Mohamad Dawelbeat, Sara Hamza Nurelhuda Sulieman, Zobida Alzubair Nasr Hussain, Kholoud Mohammed Alameen Awad Mohammed Fouad, Islam Abdalla Hussein Marmar, Iman Tarig Abdelmohsin Omer, Noha Abdelhalim Siddig Rikabi, Abubakr Muhammed, Ammar Hamad Rahmtallah

**Affiliations:** 1 Obstetrics and Gynecology, Almanagil Teaching Hospital, Almanagil, SDN; 2 Radiology, University of Cape Town, Cape Town, ZAF

**Keywords:** clinical audit, documentation standards, patient-held health records, quality improvement projects, sudan

## Abstract

Background: Maternal healthcare continuity depends on the accurate documentation, effective communication, and active participation of women in their own care. Patient-held health records, including pregnancy passports, have been proven internationally to enhance information sharing and record completeness. Nonetheless, antenatal care (ANC) documentation lapses remain a significant issue in low- and middle-income countries (LMICs) such as Sudan.

Aim: This project, developed by the Department of Obstetrics and Gynecology at Almanagil Teaching Hospital, aimed to improve the completeness of ANC documentation through the introduction of a patient-held ANC passport.

Methods: A two-cycle quality improvement project (QIP) audit was conducted during August 3-13, 2025. Cycle 1 (baseline; n=54) involved consecutive ANC records reviewed against departmental standards. This was followed by the introduction of the ANC passport, with structured staff training and patient orientation. Cycle 2 (post-intervention; n=26) again included all consecutive eligible records during the audit window. The unequal numbers between cycles reflected actual clinic attendance rather than sampling. Data were extracted using a structured checklist, and comparisons were performed using chi-squared (χ²) tests. To control for multiple comparisons, Bonferroni adjustments were applied. Analyses were conducted using IBM SPSS Statistics for Windows, Version 25.0 (Released 2019; IBM Corp., Armonk, New York, United States). Improvements were categorized as highest (≥75%), significant (50-74%), moderate (25-49%), or mild (<25%).

Findings: Baseline audit revealed major deficiencies, with many parameters undocumented (address, body mass index (BMI), bleeding history, drug and allergy history, partner name, and social history: all 0%). Post-intervention, these domains achieved 100% completeness. Significant gains were also observed for parity (0% → 96.2%), blood group (24.1% → 100%), and first-visit investigations (40.7% → 100%). Documentation of post-obstetric history improved from 0% to >50%. Most improvements were statistically significant (p<0.001).

Conclusion: Implementation of the ANC passport led to substantial improvements in the completeness of ANC documentation across nearly all parameters. This audit demonstrates that audit-driven, structured, patient-held records can enhance the accountability, continuity, and quality of ANC in resource-constrained settings. Clinical outcomes were not assessed and should be explored in future studies.

## Introduction

The continuity of maternal healthcare depends on proper documentation, good communication, and the involvement of the women in their care. Patient-held health records are increasingly becoming popular in the world, commonly known as pregnancy passports or maternal health passports, to facilitate better information exchange, greater empowerment of women, and better record completeness [1-3]. The tools offer a systematic way of monitoring the antenatal, intrapartum, and postnatal services, hence aiding in the decision-making process of the clinicians and the responsibility of the health systems.

The world has seen the introduction of a number of maternal health passport models. The Ministry of Health in Saudi Arabia created the Mother Health Passport, which is a standardized antenatal care (ANC) follow-up and education record [1]. Other programs of this kind are the Pregnancy Passport, created by the Perinatal Services British Columbia [2], to prevent the long-term risks of health conditions and the International Federation of Gynaecology and Obstetrics (FIGO) Pregnancy Passport, aimed at enhancing communication between the provider and the patient [3]. According to the reports of Saudipedia, there has been a growing appreciation of such passports as health priorities of the people [4].

Clinical audit is an already known method used to improve the quality of maternity services, especially in the low- and middle-income countries (LMICs) [5]. Africa and the Middle East have provided evidence that audits of ANC enhance compliance with the protocols, timely identification of complications, and service delivery [5,6]. A service-design model used to build the passport to maternity care has demonstrated the need to co-create with women and healthcare professionals to assure proper functionality and usefulness [7].

Almanagil Teaching Hospital in the Al-Jazirah State of Sudan is one of the major referral and teaching hospitals for the rural and semi-urban population. The need to standardize documentation tools has been emphasized by past efforts in the hospital, such as medical volunteer missions and quality improvement projects (QIPs), such as the introduction of structured investigation sheets [8-10]. Regardless of these improvements, the continuity of care and quality monitoring is constrained by the lack of continuity in the keeping of the ANC records.

The aim of this audit was thus to present and review a newly designed ANC passport, which was created through the joint efforts of the Department of Obstetrics and Gynecology at Almanagil Teaching Hospital. It was to be done to provide standardization of documentation on ANC, enhance communication between providers and pregnant women, and provide a base on which future QIP cycles could be built.

## Materials and methods

Study design and setting

This QIP clinical audit was conducted at Almanagil Teaching Hospital, a major referral and teaching hospital in the Al-Jazirah State of Sudan. The project followed a two-cycle audit design, which is the standard model for QIP initiatives, with the goal of evaluating and enhancing the completeness of ANC documentation through the introduction of a newly designed ANC passport.

Study population

The audit population consisted of ANC records of women who attended the outpatient ANC clinics at Almanagil Teaching Hospital during the two audit periods.

Inclusion and exclusion criteria

Included were all women attending ANC during the audit window with a complete ANC record available. In contrast, women with records with >50% of the ANC sheet missing, with duplicate records, or attending only for intrapartum or delivery-related visits without an ANC follow-up record were excluded from the study.

Sampling method

Records were selected consecutively in order of attendance to reflect routine clinical practice and minimize selection bias. A total of 80 records were reviewed: Cycle 1 (baseline/pre-intervention) comprised 54 records collected from August 3 to 17, 2025, while Cycle 2 (post-intervention) comprised 26 records collected from August 25 to September 13, 2025.

The difference in sample size between cycles reflected real-world clinic flow and patient attendance during the respective audit windows, rather than pre-determined or statistically powered sample selection. All eligible records within each audit period were included.

Audit standards and criteria

Audit standards were developed internally by the Department of Obstetrics and Gynecology, covering six domains of ANC documentation: (1) identifiers, (2) obstetric/gynecological history, (3) medical/social history, (4) clinical assessments, (5) investigations/ultrasound, and (6) professional accountability.

A structured checklist based on these domains was validated by two senior obstetricians and piloted before use. To ensure consistency and minimize observer bias, two trained reviewers independently extracted data, with discrepancies resolved by a third reviewer. Parameters were coded as documented/not documented to reduce subjectivity.

Cycle 1: baseline audit (August 3-17, 2025)

During the first phase, 54 ANC records were reviewed against the departmental standards using a structured checklist. This cycle identified major deficiencies in documentation, with many critical parameters absent from the records. The results provided the evidence base for targeted intervention.

Intervention (August 18-24, 2025)

**Figure 1:**
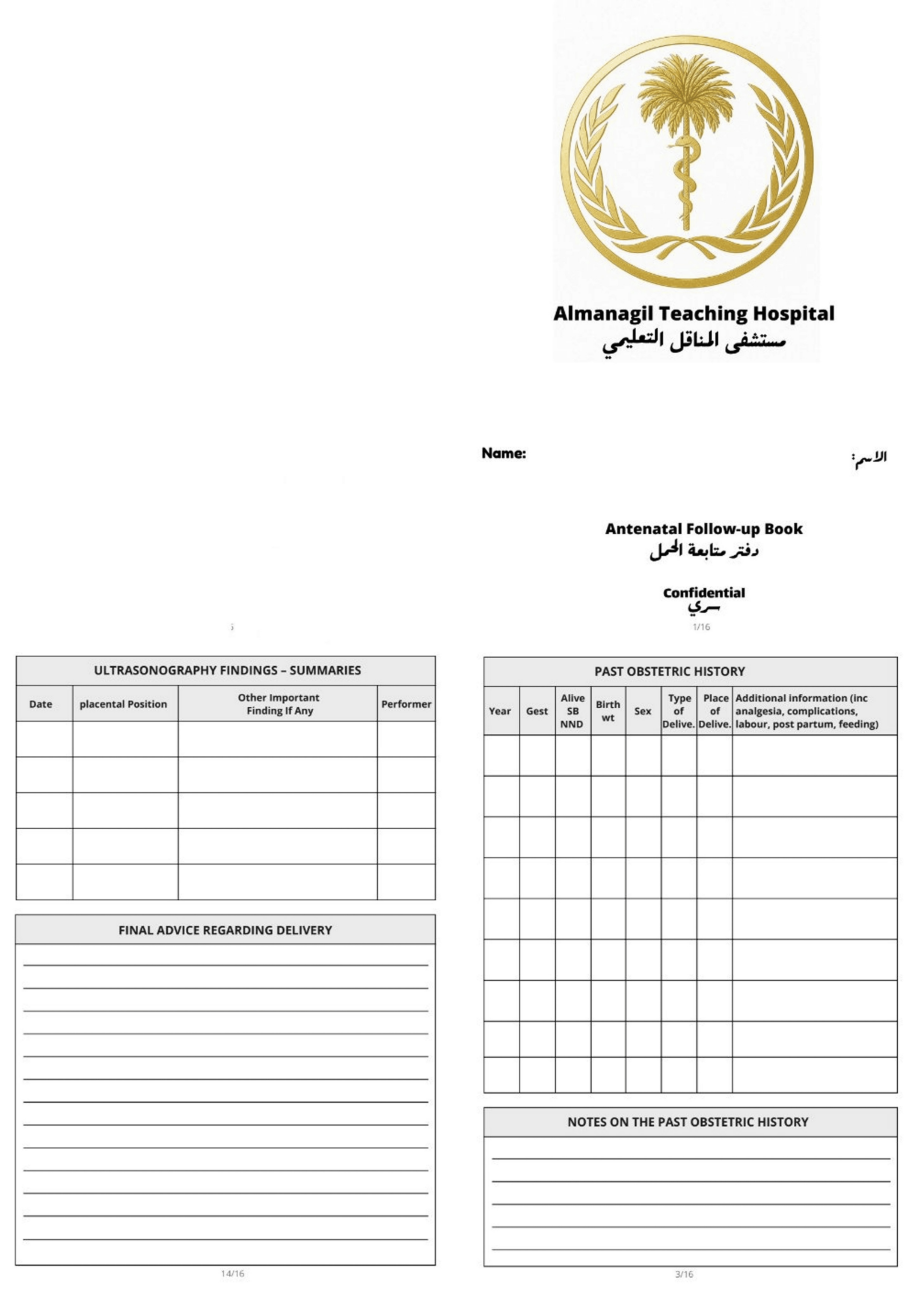
The new follow-up passport (pages 1-2)

**Figure 2:**
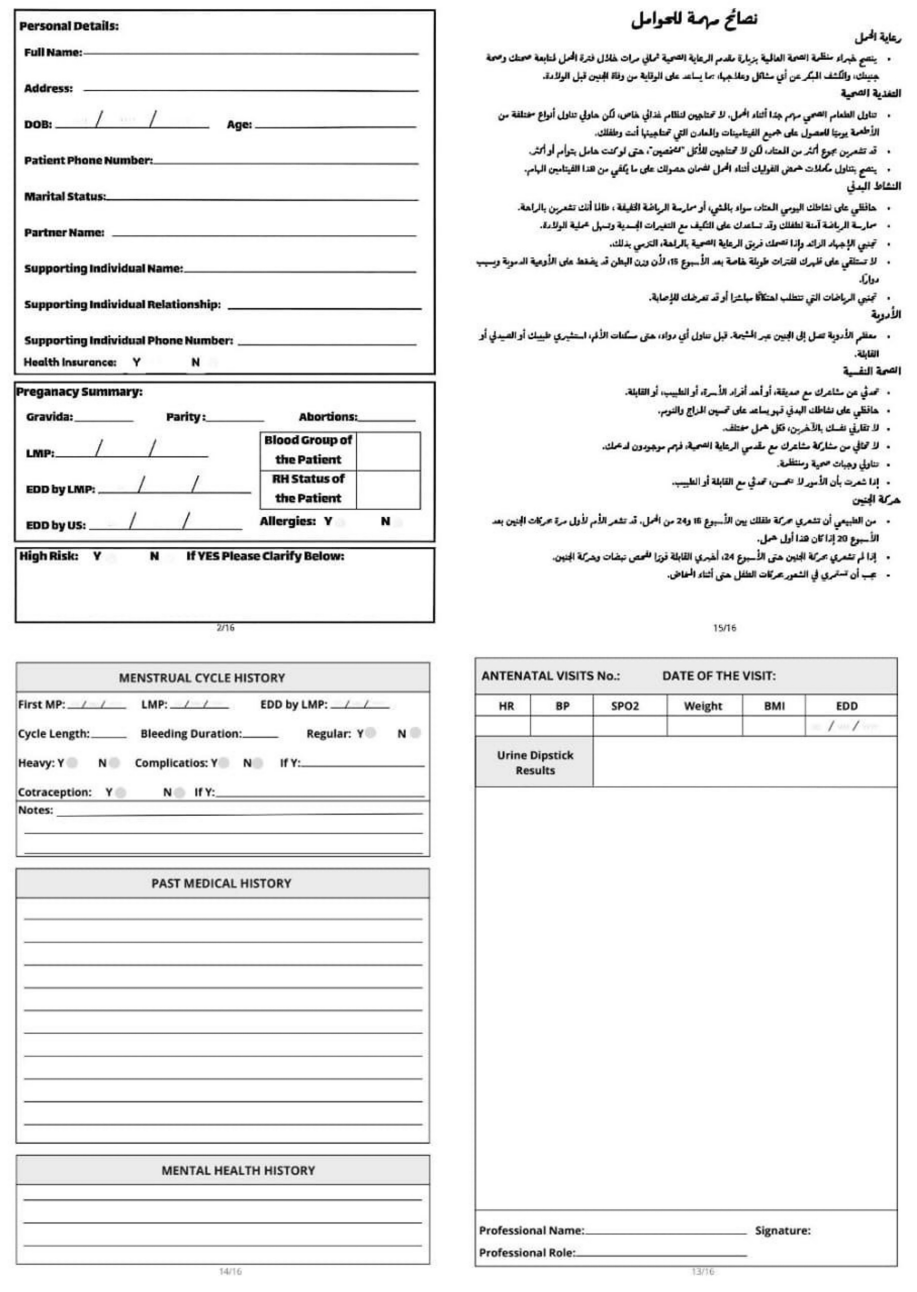
The new follow-up passport (pages 3-4)

**Figure 3:**
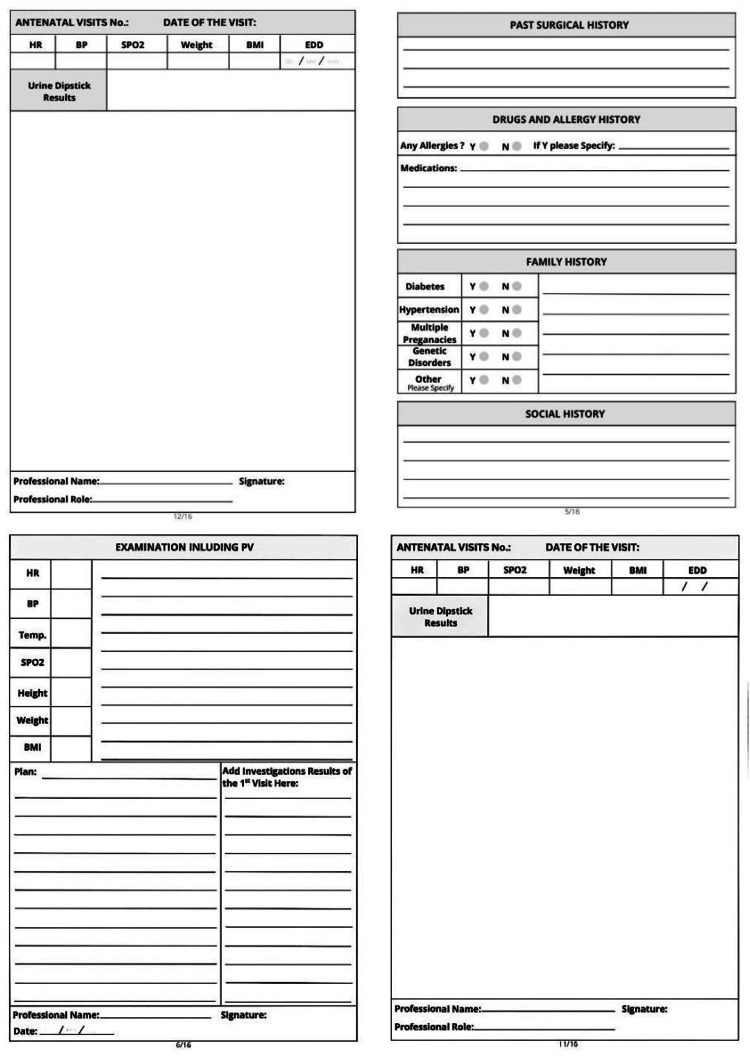
The new follow-up passport (pages 5-6)

**Figure 4:**
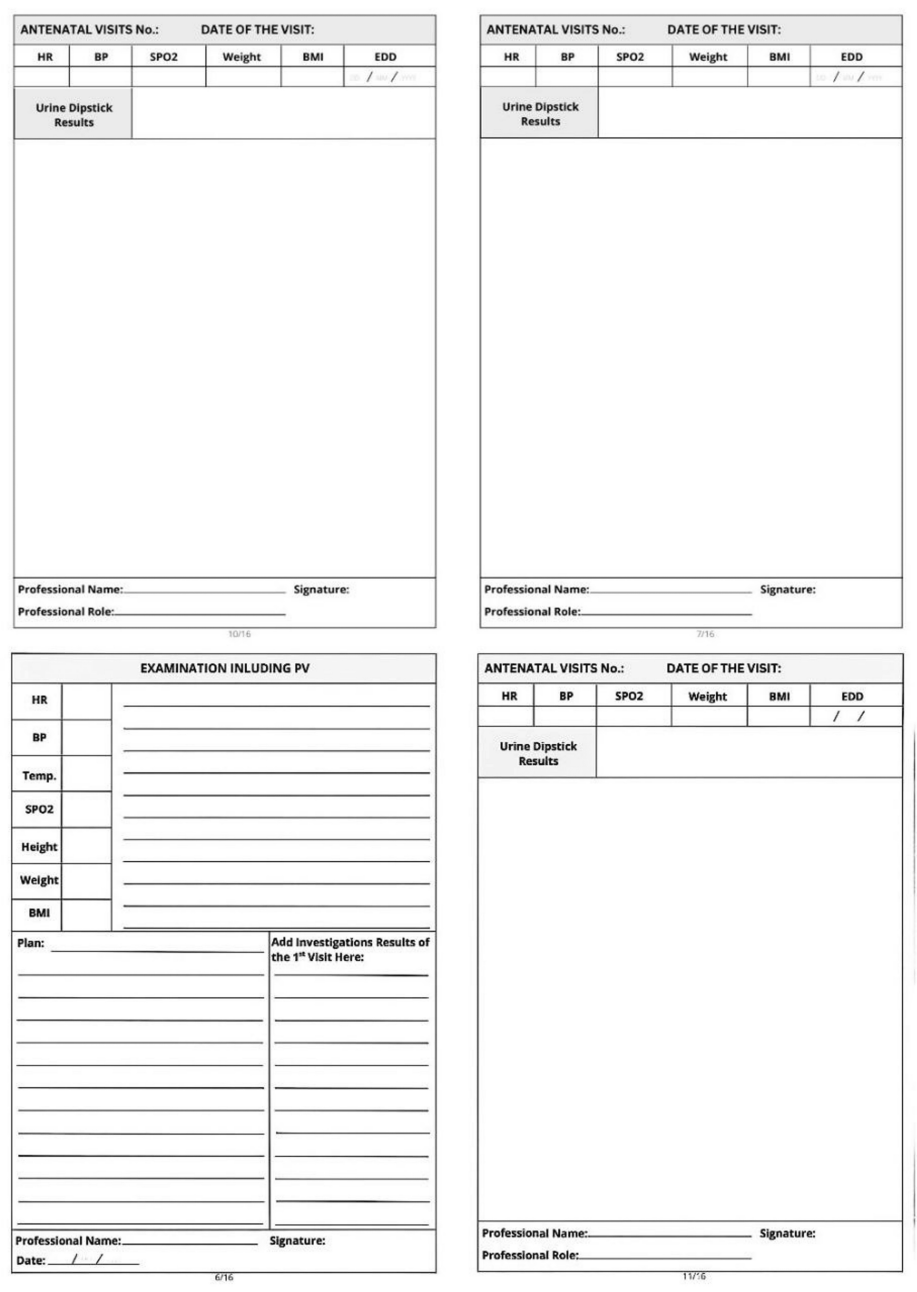
The new follow-up passport (pages 7-8)

Based on the gaps identified in Cycle 1, the ANC passport was introduced (Figures 1-4). The tool was developed collaboratively by Clinical Audit Services and the Department of Obstetrics and Gynecology to standardize record-keeping. It was designed as a patient-held document covering all essential ANC parameters.

Following the baseline audit, the ANC passport was introduced over a one-week period from August 18 to 24, 2025. During this time, four structured educational sessions were conducted: two for doctors, one for midwives, and one for nurses. Attendance was high, with approximately 95% of the ANC staff participating. In parallel, pregnant women attending the clinic were oriented on the importance of presenting and retaining their passports at each visit. To ensure adherence, supervisors performed routine spot-checks and conducted random reviews of completed passports during the re-audit period, which allowed the continuous monitoring of both staff compliance and patient engagement.

Cycle 2: re-audit (August 25 to September 13, 2025)

Following the intervention, 26 ANC records were reviewed using the same audit tool and standards. This cycle measured the effect of the ANC passport on documentation practices and determined whether improvements had been achieved.

Data collection and analysis

Data were extracted into a structured checklist aligned with departmental audit criteria, and each variable was recorded as either "documented" or "not documented". Frequencies and percentages were calculated for all parameters, and comparisons between the two audit cycles were performed using chi-squared (χ²) tests. The assumptions of the chi-squared test (expected counts ≥5 in at least 80% of cells) were verified, and Fisher's exact test was used in sensitivity analyses when assumptions were borderline. To account for multiple comparisons, Bonferroni adjustments were applied, and a two-tailed p-value of <0.05 was considered statistically significant. Improvements were then categorized as highest (≥75%), significant (50-74%), moderate (25-49%), or mild (<25%). All analyses were conducted using IBM SPSS Statistics for Windows, Version 25.0 (Released 2019; IBM Corp., Armonk, New York, United States).

Ethical considerations

The project was registered with hospital administration as a departmental QIP initiative. All patient identifiers were anonymized, and no individual consent was required under hospital and national QIP guidelines.

## Results

After the introduction of the ANC passport at Almanagil Teaching Hospital, documentation completeness improved substantially. In Cycle 1 (n=54), critical parameters such as address, body mass index (BMI), drug and allergy history, partner's name, and social history were entirely absent (0%). Clinical risk factors, including diabetes, genetic disorders, and mental health history, as well as provider accountability fields (role and signature), were also undocumented.

By Cycle 2 (n=26), universal completion (100%) was achieved in all of these areas. Other parameters, including gestational age and heart rate at first visit, improved from 1.9% to 100%, while parity, provider's name, and year reached 96-100%. Investigations at first visit, blood group, and ultrasound doctor's name rose from 16-41% to full documentation. Post-obstetric history (POH) fields, previously absent, were captured in more than half of the records, and routine physiological measures in the POH section (e.g., blood pressure (BP), weight) achieved ~46% completeness.

Chi-squared analyses confirmed that these improvements were statistically significant, with most parameters achieving p-values of <0.001 even after Bonferroni correction. Minor but consistent gains were also seen in parameters that already had high baseline completion (e.g., patient name, last menstrual period (LMP), estimated due date (EDD)).

**Figure 5:**
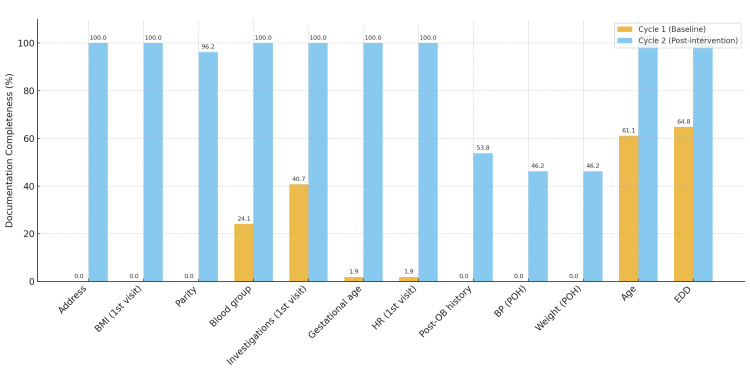
A summary of key improvements BMI: body mass index; HR: heart rate; POH: post-obstetric history; BP: blood pressure; EDD: estimated due date

A summary of key improvements is shown in Figure 5, with full parameter-level results provided in Table 1. Overall, the passport led to a marked transformation in documentation, supporting information governance, accountability, and continuity of maternal care.

**Table 1:** Comparison of documentation parameters between Cycle 1 and Cycle 2 of the antenatal care passport audit BMI: body mass index; DOB: date of birth; MP: menstrual period; LMP: last menstrual period; US: ultrasound; HR: heart rate; POH: post-obstetric history; SB: stillbirth; NND: neonatal death; EDD: estimated due date

Parameter	Cycle 1 (n=54)	Cycle 2 (n=26)	% improvement	Chi-squared	P-value	Note of improvement	Improvement category
Address	0/54 (0%)	26/26 (100%)	100	80	0.0	Improved by 100%	Highest improvement (100%)
BMI (first visit)	0/54 (0%)	26/26 (100%)	100	80	0.0	Improved by 100%	Highest improvement (100%)
Bleeding duration	0/54 (0%)	26/26 (100%)	100	80	0.0	Improved by 100%	Highest improvement (100%)
Contraception	0/54 (0%)	26/26 (100%)	100	80	0.0	Improved by 100%	Highest improvement (100%)
Cycle length	0/54 (0%)	26/26 (100%)	100	80	0.0	Improved by 100%	Highest improvement (100%)
DOB	0/54 (0%)	26/26 (100%)	100	80	0.0	Improved by 100%	Highest improvement (100%)
Diabetes	0/54 (0%)	26/26 (100%)	100	80	0.0	Improved by 100%	Highest improvement (100%)
Drug and allergy history	0/54 (0%)	26/26 (100%)	100	80	0.0	Improved by 100%	Highest improvement (100%)
First MP (menarche)	0/54 (0%)	26/26 (100%)	100	80	0.0	Improved by 100%	Highest improvement (100%)
Genetic disorders	0/54 (0%)	26/26 (100%)	100	80	0.0	Improved by 100%	Highest improvement (100%)
Heavy bleeding	0/54 (0%)	26/26 (100%)	100	80	0.0	Improved by 100%	Highest improvement (100%)
Mental health history	0/54 (0%)	26/26 (100%)	100	80	0.0	Improved by 100%	Highest improvement (100%)
Multiple pregnancies	0/54 (0%)	26/26 (100%)	100	80	0.0	Improved by 100%	Highest improvement (100%)
Partner name	0/54 (0%)	26/26 (100%)	100	80	0.0	Improved by 100%	Highest improvement (100%)
Professional role (first visit)	0/54 (0%)	26/26 (100%)	100	80	0.0	Improved by 100%	Highest improvement (100%)
Signature (first visit)	0/54 (0%)	26/26 (100%)	100	80	0.0	Improved by 100%	Highest improvement (100%)
Social history	0/54 (0%)	26/26 (100%)	100	80	0.0	Improved by 100%	Highest improvement (100%)
Weight (first visit)	0/54 (0%)	26/26 (100%)	100	80	0.0	Improved by 100%	Highest improvement (100%)
Gestational age	1/54 (1.9%)	26/26 (100%)	98.1	75.61	0.0	Improved by 98.1%	Highest improvement (98.1%)
HR (first visit)	1/54 (1.9%)	26/26 (100%)	98.1	75.61	0.0	Improved by 98.1%	Highest improvement (98.1%)
Parity	0/54 (0%)	25/26 (96.2%)	96.2	75.52	0.0	Improved by 96.2%	Highest improvement (96.2%)
Regularity	3/54 (5.6%)	26/26 (100%)	94.4	67.74	0.0	Improved by 94.4%	Highest improvement (94.4%)
Professional name (first visit)	5/54 (9.3%)	26/26 (100%)	90.7	60.88	0.0	Improved by 90.7%	Highest improvement (90.7%)
Year	5/54 (9.3%)	26/26 (100%)	90.7	60.88	0.0	Improved by 90.7%	Highest improvement (90.7%)
Past medical history	7/54 (13%)	26/26 (100%)	87	54.86	0.0	Improved by 87%	Highest improvement (87%)
Ultrasound doctor's name	9/54 (16.7%)	26/26 (100%)	83.3	49.52	0.0	Improved by 83.3%	Highest improvement (83.3%)
Blood group (patient)	13/54 (24.1%)	26/26 (100%)	75.9	40.49	0.0	Improved by 75.9%	Highest improvement (75.9%)
Investigations at the first visit	22/54 (40.7%)	26/26 (100%)	59.3	25.68	0.0	Improved by 59.3%	Significant improvement (59.3%)
Additional POH information	0/54 (0%)	14/26 (53.8%)	53.8	35.24	0.0	Improved by 53.8%	Significant improvement (53.8%)
Alive/SB/NND (POH)	0/54 (0%)	14/26 (53.8%)	53.8	35.24	0.0	Improved by 53.8%	Significant improvement (53.8%)
Place of delivery (POH)	0/54 (0%)	14/26 (53.8%)	53.8	35.24	0.0	Improved by 53.8%	Significant improvement (53.8%)
Sex (POH)	0/54 (0%)	14/26 (53.8%)	53.8	35.24	0.0	Improved by 53.8%	Significant improvement (53.8%)
Type of delivery (POH)	0/54 (0%)	14/26 (53.8%)	53.8	35.24	0.0	Improved by 53.8%	Significant improvement (53.8%)
BP (first visit)	28/54 (51.9%)	26/26 (100%)	48.1	18.55	0.0	Improved by 48.1%	Significant improvement (48.1%)
BP (POH)	0/54 (0%)	12/26 (46.2%)	46.2	29.32	0.0	Improved by 46.2%	Significant improvement (46.2%)
Date of the visit (POH)	0/54 (0%)	12/26 (46.2%)	46.2	29.32	0.0	Improved by 46.2%	Significant improvement (46.2%)
Professional role (POH)	0/54 (0%)	12/26 (46.2%)	46.2	29.32	0.0	Improved by 46.2%	Significant improvement (46.2%)
Signature (POH section)	0/54 (0%)	12/26 (46.2%)	46.2	29.32	0.0	Improved by 46.2%	Significant improvement (46.2%)
Weight (POH)	0/54 (0%)	12/26 (46.2%)	46.2	29.32	0.0	Improved by 46.2%	Significant improvement (46.2%)
SpO2 (POH)	0/54 (0%)	11/26 (42.3%)	42.3	26.49	0.0	Improved by 42.3%	Significant improvement (42.3%)
Urine dipstick (POH)	0/54 (0%)	11/26 (42.3%)	42.3	26.49	0.0	Improved by 42.3%	Significant improvement (42.3%)
Age	33/54 (61.1%)	26/26 (100%)	38.9	13.71	0.0002	Improved by 38.9%	Moderate improvement (38.9%)
BMI (POH)	4/54 (7.4%)	12/26 (46.2%)	38.7	16.47	0.0	Improved by 38.7%	Moderate improvement (38.7%)
EDD (POH)	4/54 (7.4%)	12/26 (46.2%)	38.7	16.47	0.0	Improved by 38.7%	Moderate improvement (38.7%)
Professional name (POH)	4/54 (7.4%)	12/26 (46.2%)	38.7	16.47	0.0	Improved by 38.7%	Moderate improvement (38.7%)
EDD	35/54 (64.8%)	26/26 (100%)	35.2	12	0.0005	Improved by 35.2%	Moderate improvement (35.2%)
EDD by LMP	36/54 (66.7%)	26/26 (100%)	33.3	11.18	0.0008	Improved by 33.3%	Moderate improvement (33.3%)
Other important US findings	36/54 (66.7%)	26/26 (100%)	33.3	11.18	0.0008	Improved by 33.3%	Moderate improvement (33.3%)
Date of ultrasound	38/54 (70.4%)	26/26 (100%)	29.6	9.63	0.0019	Improved by 29.6%	Moderate improvement (29.6%)
Temperature (POH)	0/54 (0%)	4/26 (15.4%)	15.4	8.74	0.0031	Improved by 15.4%	Mild improvement (15.4%)
Height (POH)	0/54 (0%)	3/26 (11.5%)	11.5	6.47	0.0109	Improved by 11.5%	Mild improvement (11.5%)
Date of the visit (first)	48/54 (88.9%)	26/26 (100%)	11.1	3.12	0.0772	Improved by 11.1%	Mild improvement (11.1%)
LMP	48/54 (88.9%)	26/26 (100%)	11.1	3.12	0.0772	Improved by 11.1%	Mild improvement (11.1%)
BMI (later visit)	0/54 (0%)	2/26 (7.7%)	7.7	4.26	0.039	Improved by 7.7%	Mild improvement (7.7%)
BP (later visit)	0/54 (0%)	2/26 (7.7%)	7.7	4.26	0.039	Improved by 7.7%	Mild improvement (7.7%)
Date of the visit (later)	0/54 (0%)	2/26 (7.7%)	7.7	4.26	0.039	Improved by 7.7%	Mild improvement (7.7%)
HR (later visit)	0/54 (0%)	2/26 (7.7%)	7.7	4.26	0.039	Improved by 7.7%	Mild improvement (7.7%)
SpO2 (later visit)	0/54 (0%)	2/26 (7.7%)	7.7	4.26	0.039	Improved by 7.7%	Mild improvement (7.7%)
Urine dipstick (later)	0/54 (0%)	2/26 (7.7%)	7.7	4.26	0.039	Improved by 7.7%	Mild improvement (7.7%)
Weight (later visit)	0/54 (0%)	2/26 (7.7%)	7.7	4.26	0.039	Improved by 7.7%	Mild improvement (7.7%)
Gravida	48/54 (88.9%)	25/26 (96.2%)	7.3	1.16	0.2814	Improved by 7.3%	Mild improvement (7.3%)
Name	53/54 (98.1%)	26/26 (100%)	1.9	0.49	0.485	Improved by 1.9%	Mild improvement (1.9%)

## Discussion

This QIP also showed that the implementation of a newly designed ANC passport, which was collaboratively designed by the Department of Obstetrics and Gynecology of Almanagil Teaching Hospital in the Al-Jazirah State of Sudan, resulted in significant changes in the completeness of ANC documentation. The results prove that the intervention, based on targeted and audit-focused cycles, can change the conditions of maternal health record-keeping in resource-constrained environments.

The identified documentation misgap observed in the process of the baseline audit is not unique to the country since it has been noted that the documentation misgap is similar to that of other LMICs where ANC records are generally incomplete, fragmented, or unavailable [4-6]. The post-intervention gains that have been large are consistent with global experiences in the use of pregnancy passports and home-based maternal health records. The Saudi Mother Health Passport, the Pregnancy Passport in British Columbia, and the FIGO Pregnancy Passport, among others, were all aimed at enhancing the continuum of dialogue between women and providers and the standardization of ANC documentation [1-3]. Evidence regarding the use of maternal health passports is provided by systematic reviews demonstrating the increased completeness of documentation and maternal involvement in care [4]. Equally, research in Africa has revealed that clinical audits enhance compliance with protocols and improve antenatal service provision [5,6]. These trends are supported by the results of this audit and provide new evidence from Sudan, which represents one of the earliest reported attempts of this type in the region.

The major missing items, which were pointed out in Cycle 1 of the audit, comprised key identifiers and risk factors such as diabetes, genetic disorders, and mental health history, along with basic clinical information such as BP, BMI, and partner data. Cycle 2, after the implementation of the ANC passport, demonstrated completeness across all categories that were statistically significant across most parameters (p<0.001). Improvements were not limited to individual areas but also extended to systematic recording of medical, obstetric, and social history, as well as professional accountability measures such as provider name, role, and signature. The consistent monitoring of physiological parameters, including BP, weight, heart rate, and oxygen saturation, proves the clinical usefulness of the passport in continuous maternal monitoring. The formatted design of the passport ensured that the necessary parameters were fully covered and that the chances of omissions were diminished. Staff training and the patient-held format promoted behavioral change and shared responsibility for record-keeping between the provider and the woman.

The implementation of standardized ANC records in one of the major referral hospitals located in Al-Jazirah State has significant meaning to Sudanese maternal healthcare. The passport solves the long-standing problem of discontinuity between information held by patients and by providers [7,9]. Its portability is of great importance, especially in rural and semi-urban areas where women may visit various providers or facilities. Co-creation with frontline staff proved valuable, as the service-design model outlined by Salgado et al. [7] stresses practicality, acceptability, and ownership. This partnership approach aided the adoption and integration of the tool into routine clinical practice. Moreover, the effectiveness of such interventions in this project is also featured, which is why such interventions can be scaled to other settings where electronic health records are not yet common.

Given the success of this departmental initiative at Almanagil Teaching Hospital in the Al-Jazirah State of Sudan, future QIP cycles could be expanded to other hospitals and regions across the country. A multi-center approach would allow the development of standardized national protocols and clear documentation guidelines to improve ANC quality at the national level.

One of the strongest points of this audit was that it is in line with departmental priorities and utilized the two-cycle audit model that allowed benchmarking and reliable measurement of improvement. The success of the intervention is supported by universal or near-universal gains across many parameters. Nevertheless, the post-intervention sample size (Cycle 2; n = 26) is relatively small, which restricts generalizability and does not reflect long-term sustainability. In addition, the audit targeted only documentation completeness and did not determine maternal or neonatal outcomes. This should be addressed in future cycles with larger cohorts and longer follow-ups to establish whether the observed changes are sustainable and translate into better clinical outcomes.

This audit has several limitations. First, potential selection bias may have arisen because record inclusion depended on clinic attendance during the audit windows, which may not represent all ANC attendees. The post-intervention sample size in Cycle 2 (n=26) was relatively small, which limits generalizability. Second, although dual independent reviewers and supervisory checks were used, the same team that implemented the passport also conducted the audit, introducing possible observer bias.

The audit measured documentation completeness only and did not evaluate maternal or neonatal outcomes; hence, the study assessed internal improvement in process quality but not external clinical impact. The follow-up period was short, and the long-term sustainability of the intervention remains untested. Finally, as the audit was conducted in a single teaching hospital, external validity is limited. Future multi-center replication, including facilities in other Sudanese states, would help confirm the transferability of results and inform national guideline development.

Despite these constraints, the methodological transparency, structured design, and strong internal consistency support the reliability of the findings and provide a practical framework for replication in similar low-resource contexts.

This audit shows how methodical documentation tools incorporated into a clinical audit system can increase and enhance the keeping of ANC records more efficiently. Large-scale implementation of such passports, building on the experience at Almanagil Teaching Hospital, could support national maternal health registries, standardize monitoring, and enhance maternal outcomes across Sudan. Future studies should track the sustainability of the passport's use, explore women's experiences, extend its application to intrapartum and postnatal stages, and examine how it can be integrated with digital health approaches to optimize its impact.

## Conclusions

The introduction of the ANC passport at Almanagil Teaching Hospital in the Al-Jazirah State of Sudan resulted in a marked improvement in the completeness of ANC documentation across nearly all parameters. These findings demonstrate that structured, audit-based interventions can strengthen information governance, accountability, and continuity of care in resource-constrained settings. Building on the success achieved at Almanagil Teaching Hospital, future QIP cycles could be expanded to other hospitals and regions across Sudan. Such a multi-center implementation would help establish standardized national protocols and clear documentation guidelines to enhance the quality and consistency of ANC nationwide. However, the results should be interpreted in light of the study's limitations, and future work with larger samples, longer follow-up, and incorporation of clinical outcome measures is required to determine the broader impact on maternal and neonatal health.
